# Pregnancy and delivery in women receiving maintenance hemodialysis in Japan: analysis of potential risk factors for neonatal and maternal complications

**DOI:** 10.1007/s40620-021-01146-3

**Published:** 2021-09-30

**Authors:** Hiroko Hirano, Tomomi Ueda, Hirohiko Tani, Kenzo Kosaka, Eiji Nakatani, Philip Hawke, Kiyoshi Mori, Noriko Mori

**Affiliations:** 1 Department of Nephrology, Shizuoka General Hospital, 4-27-1 Kita-ando, Aoi-ku, Shizuoka, 420-8527 Japan; 2 Department of Obstetrics and Gynecology, Shizuoka General Hospital, Shizuoka, Japan; 3 Graduate School of Public Health, Shizuoka Graduate University of Public Health, Shizuoka, Japan; 4 Research Support Center, Shizuoka General Hospital, Shizuoka, Japan; 5 Laboratory of Scientific English, School of Pharmaceutical Sciences, University of Shizuoka, Shizuoka, Japan; 6 Department of Molecular and Clinical Pharmacology, School of Pharmaceutical Sciences, University of Shizuoka, Shizuoka, Japan

**Keywords:** Delivery, Dialysis, Complication, Pregnancy, Preterm

## Abstract

**Introduction:**

Average dialysis vintage in Japan is among the longest in the world, providing a unique opportunity to characterize pregnancy under conditions of long dialysis vintage. In 2017, we carried out a nationwide survey following up on a similar survey in 1996, in which we investigated the prevalence and outcomes of pregnancy in women undergoing dialysis and assessed risk factors associated with neonatal and maternal complications.

**Methods:**

The target population was women aged 15–44 years undergoing maintenance dialysis between 2012 and 2016. The survey was conducted in 2693 dialysis units.

**Results:**

A response was obtained from 951 dialysis units, yielding a target population of 1992 women of childbearing age receiving hemodialysis or peritoneal dialysis. Pregnancy occurred only among women receiving hemodialysis, with 25 pregnancies (1.26% in 5 years) being reported for 20 women. Detailed information about 19 pregnancies (mean age 34.6 ± 5.7 years at conception, mean dialysis vintage 8.4 ± 7.3 years) indicated 4 spontaneous abortions, 1 elective abortion, no neonatal deaths, and 14 surviving infants, including 5 full-term (≥ 37 weeks at birth), 2 late preterm (34–36), and 3 extremely preterm (< 28) cases. Neonatal complications occurred in the offspring of 3 mothers who had end-stage renal disease (ESRD) caused by primary glomerulonephritis and serum albumin levels (sAlb) ≤ 3.2 mg/dL in the first trimester. These mothers had started dialysis at 12, 17, and 30 years of age. ESRD caused by diabetic nephropathy or primary glomerulonephritis, age at conception ≥ 38 years, and sAlb ≤ 3.2 mg/dL were associated with maternal complications, although not significantly.

**Conclusions:**

In this study, the pregnancy rate of Japanese women with ESRD was 0.25% per year. The study generates the hypothesis that ESRD caused by diabetic nephropathy and age at conception ≥ 38 years are potential risk factors for maternal complications but not for neonatal complications in dialysis patients, and that hypoalbuminemia is a potential risk factor for both kinds of complications.

**Graphic Abstract:**

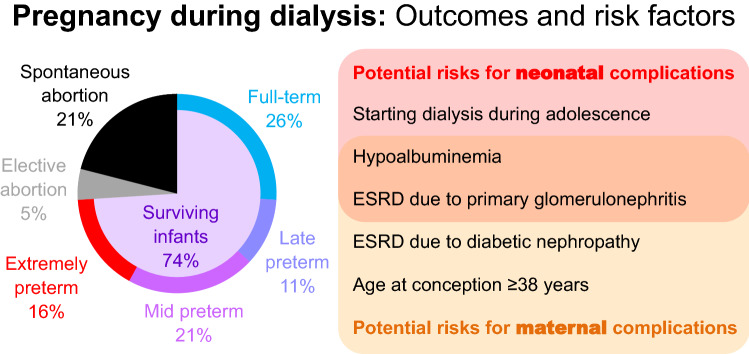

**Supplementary Information:**

The online version contains supplementary material available at 10.1007/s40620-021-01146-3.

## Introduction

Compared to the general population and to renal transplant patients, women with end-stage renal disease (ESRD) undergoing dialysis are at greater risk of infertility, fetal death in early pregnancy period, and premature birth [1]. However, renal transplantation is difficult to access in Japan because of the limited number of cadaveric organ donations. In 2016, the average waiting time for cadaveric renal transplantation was 15.5 years [2]. The number of occurrences of living and cadaveric renal transplantation were 1471 and 177, respectively [3] (only 4.0% of total ESRD patients), whereas 39,344 patients started hemodialysis or peritoneal dialysis in the same year [4]. According to the Dialysis Outcomes and Practice Patterns Study, around the year 2000, the mean number of years on dialysis was 7.4 years in Japan, 5.1 years in Europe, and 3.4 years in the USA [5]. In Japan, the health insurance system covers most of the cost of dialysis treatment, which has enabled intensive treatment. As dialysis access and vintage are much greater in Japan than in other countries, the Japanese situation provides a unique opportunity to study pregnancy under long-term dialysis.

The first ever description of conception and successful delivery in a woman with ESRD was reported in 1971 [6]. In Japan, the first successful delivery was achieved in 1977 [7]. A nationwide survey by Toma et al. in 1996 reported that the frequency of pregnancy in Japanese dialysis patients was 3.4% and the live birth rate, excluding cases with elective abortion and unknown outcome, was 66.7% [8]. A retrospective study at a single Japanese dialysis unit in 2009 reported a similar live birth rate of 64.3% [9]. According to recent surveys in other countries, higher live birth rates in hemodialysis patients are associated with decreased blood urea nitrogen (BUN) levels caused by high frequency dialysis [9–11]. Since 1996, there had been no nationwide survey about pregnancy in women with ESRD undergoing dialysis in Japan. Therefore, we undertook a survey 21 years later to update the information on the dialysis settings and outcomes. Furthermore, we collected blood pressure (BP) and biochemical data during each trimester for women undergoing dialysis, something that had not been well described in previous reports. These findings may help to improve the outcomes of pregnancy and delivery among women under dialysis. This study was carried out under the name of the Tsubasa Project, as one of the studies on gender led by young Japanese female doctors and supported by The Japanese Society for Dialysis Therapy (JSDT).

## Materials and methods

### Study population and design

This study was a nationwide, multi-unit, retrospective survey carried out in 2017. The target population was women aged 15–44 years with ESRD undergoing dialysis [8] between January 1, 2012 and December 31, 2016 in Japan.

### Two-step survey

A preliminary questionnaire was sent by mail to the directors of all dialysis units in Japan who were taking care of at least one patient in the target population. In this preliminary study, information was collected concerning the number of women with ESRD and those who became pregnant in that period. The applicable dialysis units were determined according to the annual JSDT survey carried out in December 2016.

Based on the response to the first questionnaire from the dialysis units, more detailed information was collected by the second questionnaire, including the following items: nature of the underlying renal disease, length of time on dialysis, mode and dose of hemodialysis, obstetric course, maternal and neonatal complications including hypertensive disorders of pregnancy (HDP), premature labor, polyhydramnios, and need for cesarian section. Predialysis biochemical parameters at the beginning of the week, dry weights, and BP at around 12–14, 20–22, and 32–34 weeks of gestation were collected.

### Outcomes

**Fig. 1:**
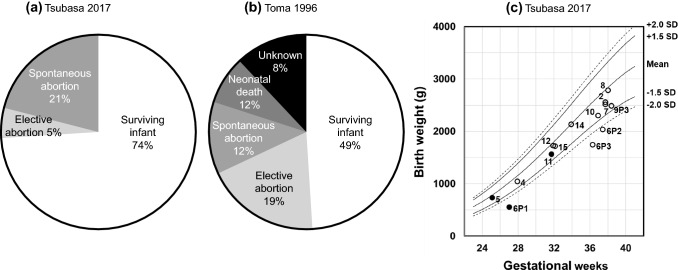
Outcomes of pregnancy in women undergoing hemodialysis in Japan, and association between birth weight, gestational age and neonatal complication. Comparison between the current study (**a**) and the previous study (**b**) is shown in pie charts. **b** was reproduced and modified from Toma et al. [8] by permission. **c** The gestational ages and birth weights of 14 live births in this study were tightly associated (*P* < 0.001, *R* = 0.94). The presence or absence of neonatal complications is indicated by black (n = 3) and white dots (n = 11), respectively. Japanese reference values (mean, ± 1.5 SD, and ± 2.0 SD) [12] and case numbers are also shown

Outcomes were defined as follows: *surviving infants*, infants who were alive for more than 28 days [8, 9]; *neonatal death*, death within the first 28 days of life [8, 9]; *intrauterine fetal demise*, intrauterine fetal death after the first trimester and before the onset of labor; *spontaneous abortion*, loss of pregnancy without artificial intervention before 22 weeks of gestation (according to the definition by the Japan Society of Obstetrics and Gynecology); *elective abortion*, termination of pregnancy because of anticipated medical problems or social reasons; and *fetal growth restriction* (*FGR*), fetal weight smaller than − 1.5 standard deviation (SD) of Japanese reference value (Fig. 1) [12]. *HDP* was defined as systolic BP of 140 mmHg or higher, diastolic BP of 90 mmHg or higher, or use of antihypertensive drugs [13]. BP ≥ 160/110 mmHg was defined as *severe hypertension*. Gestational ages were categorized as full-term (≥ 37 weeks), late preterm (34–36 weeks) [14], and extremely preterm (< 28 weeks) [15].

### Statistical analyses

The data were summarized as frequency for categorical variables and means ± SD for continuous variables. A normality test was not carried out. If multiple pregnancies occurred in a single patient, midpoint was used to calculate mean age at conception and mean duration of hemodialysis before pregnancy. Groups were compared using an unpaired t-test for continuous variables and a chi-square test or Fisher’s exact test for categorical variables. To study the association between birth weight and gestational age at delivery, a univariate linear regression analysis was performed. To determine the cutoff level for serum albumin to predict pregnancy complications, a receiver operating characteristic (ROC) curve analysis was carried out using GraphPad Prism (MDF, Tokyo, Japan). Adjustment of *P* values for multiple comparison was not performed, since this was not a confirmatory but a descriptive study. *P* < 0.05 was considered statistically significant. Statistical analysis was performed using SPSS (Chicago, IL, USA).

## Results

### Frequency of pregnancy

A preliminary questionnaire was provided to all 2693 dialysis units in Japan treating at least one woman of childbearing age, to which 951 units responded (response rate 35.3%). Among a target population of 1992 in those units (covering 27.6% of such women in Japan), 25 pregnancies (1.26%/5 years) were reported in 20 women during the years 2012 to 2016 (Fig. S1). No maternal death was reported. All pregnancies occurred in patients on maintenance hemodialysis, with no peritoneal dialysis cases being reported.

### Live birth rate

Under written informed consent, more detailed information was obtained on 19 pregnancies (76.0% of 25) in 15 patients (Fig. S1). There were 4 spontaneous abortions (21.1%, at ages 21, 30, 35 and 38), 1 elective abortion (5.3%), 14 surviving infants (73.7%), no intrauterine fetal demise and no neonatal deaths (Fig. 1). There were 9 preterm and 5 full-term deliveries. The live birth rate was 77.8%, excluding the case of elective abortion. Spontaneous abortions occurred at mean of 8.5 weeks of gestation. The elective abortion was performed in the 9th week. In women on dialysis for more than 10 years, 7 pregnancies (36.8% of total pregnancies) were reported (Table 1). They resulted in 5 surviving infants (71.4%) and 2 spontaneous abortions (28.6%).

**Table 1:** Maternal characteristics, outcome of pregnancy and neonatal complications in pregnancy on hemodialysis

	Age at dialysis initiation/conception (years)	Dialysis vintage (years)	Cause of ESRD	cHT	sAlb (mg/dL)	Maternal complication	Hospitalization (weeks)/delivery	Reason for CS	Outcome	GA (weeks)	BW (g)	BW (SD)	Neonatal complication
1	20/21	0.8	Alport		2.9	None	–	–	S. abortion	–	–		–
2	24/26	2.4	PKD		3.7	None	37/CS	Recommended by a doctor	Surviving	37 + 5	2562	− 1.0	None
3	29/30	0.8	DN	+	3.8	HDP	–	–	S. abortion	–	–		–
4	17/32	15.1	Alport		3.6	None	23/CS	NRFS	Surviving	27 + 6	1048	− 1.2	None
5	12/32	20.5	NS		3.2	HDP (sHT), cervical insufficiency, preterm labor	19/V	–	Surviving	25 + 1	733	− 1.1	RDS, rickets
6 P1	30/33	2.8	CGN	+	3.2	HDP	20/CS	FGR	Surviving	27 + 0	555	− 3.9	Retinopaty, rickets, ASD, VSD
6 P2	30/34	4.3		+	3.1	HDP	29/CS	After CS	Surviving	37 + 3	2038	− 2.4	None
6 P3	30/36	5.8		+	3.2	HDP (sHT)	32/CS	After CS	Surviving	36 + 2	1750	− 3.0	None
7	26/35	9.1	PurpN		3.9	Cervical insufficiency	20/CS	NRFS	Surviving	37 + 5	2520	− 1.1	None
8	28/35	7.4	InterstN		3.6	None	38/V	–	Surviving	38 + 0	2788	− 0.4	None
9 P1	22/35	12.6	IgAN		3.4	None	–	–	S. abortion	–	–		–
9 P2	22/38	15.0			3.7	None	–	–	S. abortion	–	–		–
9 P3	22/38	15.1			3.6	HDP	30/V	–	Surviving	38 + 3	2490	− 1.5	None
10	35/38	3.4	DN	+	3.7	HDP (sHT)	20/CS	PROM, sHT	Surviving	36 + 6	2303	− 1.4	None
11	17/39	21.8	MPGN		3.2	Total placenta previa, preterm labor	22/CS	Total placenta previa, placental abruption	Surviving	31 + 5	1564	− 1.4	RDS, cerebral palsy, epilepsy, neonatal jaundice, primary glaucoma
12	25/40	15.2	ReflN		3.3	HDP (sHT), preterm labor	22/V	–	Surviving	31 + 6	1728	− 0.7	None
13	39/40	1.0	PKD		3.7	None	–	–	E. abortion	–	–		–
14	32/40	7.7	DN		3.3	HDP, cervical insufficiency, ileus, preterm labor	20/CS	Ileus	Surviving	33 + 6	2138	− 0.4	None
15	38/40	2.3	DN	+	3.4	HDP (sHT), cervical insufficiency, preterm labor	21/CS	NRFS	Surviving	32 + 1	1720	– 0.7	None

*cHT* chronic hypertension at pregnancy start, *sAlb* serum albumin at the first trimester, *CS* cesarean section, *GA* gestational age shown as weeks + days, *BW* birth weight, *P1–P3* the first, second and third pregnancy, *Alport* Alport syndrome, *PKD* polycystic kidney disease, *IgAN* IgA nephropathy, *DN* diabetic nephropathy, *MPGN* membranoproliferative glomerulonephritis, *PurpN* purpura nephritis, *Refl* Reflux nephropathy, *NS* nephrotic syndrome, *CGN* chronic glomerulonephritis, *InterstN* interstitial nephritis, *V* vaginal delivery, *HDP*, hypertensive disorders of pregnancy; *sHT*, severe hypertension during pregnancy; *NRFS* non-reassuring fetal status, *FGR* fetal growth restriction, *PROM* pre-mature rupture of membranes, *Surviving* surviving infant, *S. abortion* spontaneous abortion, *E. abortion* elective abortion, *RDS* respiratory distress syndrome, *ASD* atrial septal defect, *VSD* ventricular septal defect

### Characteristics and outcomes of the mothers

Among the 15 women who became pregnant during the study period, 13 became pregnant once, and 2 became pregnant 3 times (Tables 1, S1). Mean age at conception was 34.6 ± 5.7 (range 21–40, n = 15) years. Mean duration of hemodialysis before pregnancy was 8.4 ± 7.3 (range 0.8–21.8, n = 15) years. In the third trimester, mean dialysis time and frequency were 26.3 ± 5.1 h and 5.6 ± 0.5 times per week (n = 10), respectively (Table S2). Before delivery, all pregnant women were admitted to hospital at mean of 25.2 ± 6.7 weeks (n = 14) to undergo more frequent dialysis, to maintain close maternal and fetal monitoring, or to respond to the onset of pregnancy complications. Maternal complications occurred in 12 pregnancies out of 19 (63.2%). There were 6 cases with chronic hypertension at pregnancy start (31.6%). HDP was observed in 10 cases (52.6%), including a case of spontaneous abortion. Five of these had severe hypertension, and all 5 had preterm deliveries. Among 6 women who became pregnant at 38 years of age or older and delivered surviving infants, 5 experienced HDP, and 5 had preterm deliveries (Tables 1, S1). Maternal complications other than HDP included cervical insufficiency, total placenta previa, and preterm labor. Overall modes of delivery included 4 vaginal deliveries and 10 cesarean sections. The reasons for preterm delivery and cesarean section are shown in Table 1. No polyhydramnios was reported.

### Biochemistry, anthropometry findings and dialysis prescription in trimesters

Biochemical parameters, BP, dry weight and dialysis prescription in each trimester are shown in Table S2 (n = 10–19). Modification of the dialysis prescription began at 11 ± 6.6 weeks of pregnancy. Among 19 pregnancies, the mean dialysis prescription was 18.4 ± 6.6 h and 4.0 ± 1.0 times per week in the first trimester (at 10.8 ± 3.1 weeks). BUN, creatinine, potassium, and phosphate levels decreased as the trimester progressed. Hemoglobin levels were kept intact around 10 g/dL. Dry weights increased by a mean of 0.19 ± 0.15 kg/week between 12.3 and 20.9 weeks (n = 14), with an increase rate of 0.24 ± 0.21 kg/week between 20.8 and 32.6 weeks (n = 10).

### Detailed data on delivery and children

All of the 14 newborn infants lived for more than 28 days. Mean gestational age at delivery was 33.7 ± 4.5 weeks (Table 1). Mean birth weight was 1853 ± 694 g. Eleven (78.6%) of the 14 infants were below 2500 g in birth weight. Eight had low birth weights (1500–2500 g), 1 had very low birth weight (1000–1500 g), and 2 had extremely low birth weights (< 1000 g). These 2 extremely low birth weight infants were also extremely preterm. They were among 3 infants who exhibited neonatal complications other than congenital abnormalities. The third had low birth weight and preterm delivery (31 weeks). Among 9 preterm infants, there were 2 late preterm infants. One of the 4 FGR infants who had neonatal complications had a birth weight at 27 weeks of − 3.9 SD of the Japanese reference value (555 g, Tables 1, S1). Among the 14 infants, a strong correlation was observed between gestational age and birth weight (Fig. 1, *P* < 0.001, *R* = 0.94 by Pearson rank test).

Neonatal complications occurred in 3 infants, the mothers of whom had started dialysis at 12, 17, and 30 years of age and had dialysis vintages of 20.5, 21.8, and 2.8 years, respectively. Case 5 had marked changes in status from the first to the second trimesters, developing severe hypertension (BP changed from 108/73 to 175/105 mmHg) and severe malnutrition (BUN from 63 to 35 mg/dL, albumin from 3.2 to 2.2 mg/dL, and phosphate from 5.1 to 2.5 mg/dL). Case 6 had chronic hypertension at pregnancy start and delivered 3 living infants, all of whom had FGR, suggesting that a persistent maternal problem existed. Case 11 developed placental abruption due to total placenta previa. Regular use of anticoagulants for dialysis and presence of borderline FGR (− 1.4 SD) might have caused a worsening of neonatal complications in this infant.

### Potential risk factors for neonatal and maternal complications associated with surviving infants

To highlight potential risks for adverse outcomes of pregnancy and delivery, risks were graded as shown in Table S1, as a color-coded version of Table 1. Among the 14 surviving infants, neonatal complications occurred among 3 mothers who had primary glomerulonephritis (PGN; nephrotic syndrome, chronic glomerulonephritis, or membranoproliferative glomerulonephritis) as the cause of ESRD (3/7 = 25.1% vs 0/7 = 0% with other causes of ESRD, not significant). Moreover, serum albumin level (sAlb) ≤ 3.2 mg/dL in the first trimester (3/5 = 60.0%, Fig. S2) was significantly associated with increased incidence of neonatal complications (vs 0/9 = 0% with ≥ 3.3 mg/dL, *P* = 0.03).

Concerning maternal complications, the following tendencies were observed. As to the cause of ESRD, diabetic nephropathy or PGN (10/10 = 100%) was associated with increased incidence of maternal complications (vs 1/4 = 25% with other causes, *P* = 0.01). Age at conception ≥ 38 years (6/6 = 100%) was associated with increased incidence of maternal complications (vs 5/8 = 62.5% with ≤ 37 years), but not with neonatal complications (1/6 = 16.7% vs 2/8 = 25.0%). First trimester sAlb ≤ 3.2 mg/dL (5/5 = 100.0%, Fig. S2) was associated with maternal complications (vs 6/9 = 66.7% with ≥ 3.3 mg/dL). Dialysis vintage ≥ 10 years (4/5 = 80.0%) was not associated with maternal complications (vs 7/9 = 77.8% with < 10 years). Chronic hypertension at pregnancy start was a major component of maternal complications (6 out of 11), but its presence was not associated with neonatal complications (1/5 = 20.0% vs 2/9 = 22.2% with no chronic hypertension).

### Comparison between preterm and full-term deliveries

As shown in Table 1, compared to the 5 full-term cases, the 9 preterm cases had significantly lower birth weights (preterm 1504 ± 603 g versus full-term 2480 ± 273 g, *P* = 0.005), earlier gestational weeks at hospitalization (22.1 ± 3.9 versus 30.8 ± 7.3 weeks, *P* = 0.01), and developed HDP more frequently (77.8% versus 40.0%, *P* = 0.16). Among women who reached the third trimester (n = 10), systolic (141 ± 21 versus 115 ± 15 mmHg, *P* < 0.05) and diastolic BPs (90 ± 12 versus 68 ± 10 mmHg, *P* = 0.01) were significantly higher in preterm cases. During the first trimester, women who would later have a preterm delivery tended to have lower BUN, albumin, and phosphorus levels, suggesting that they had malnutrition. Furthermore, although the differences were not significant, women who had preterm deliveries were older (mean ages 36.7 ± 3.5, range 32–40 versus 33.6 ± 4.5, 26–38, *P* = 0.18) and duration of dialysis was longer (10.5 ± 7.7, 2.3–21.8 versus 7.6 ± 4.9, 2.4–15.1 years, *P* = 0.47) compared to those who had full-term deliveries. No neonatal complications were observed in full-term deliveries.

## Discussion

In the present survey of the Tsubasa Project, among 1992 Japanese women with ESRD aged 15–44 years, 25 pregnancies (1.26%/5 years) were reported for 20 women receiving hemodialysis. Detailed information about 19 pregnancies was collected for 15 women. Compared to the previous survey in 1996 [8], when cases with elective abortion and unknown outcome were excluded, the live birth rate elevated from 66.7 to 77.8% (but not significantly, *P* = 0.38). Among 14 pregnancies resulting in surviving infants, 5 mothers had undergone dialysis for more than 10 years, and 11 maternal and 3 neonatal complications occurred. Age at conception ≥ 38 years tended to have an increased association with maternal complications compared to ≤ 37 years, but neonatal complications developed in mothers whose ages were 32, 33 and 39 years. Furthermore, malnutrition suggested by sAlb ≤ 3.2 mg/dL in the first trimester appears to be a common risk for both maternal and neonatal complications [16, 17]. These complex findings are in line with a previous report by Villar et al. which proposed that preeclampsia (as a maternal complication) and FGR (as a neonatal complication) may have distinct etiologies among obstetric disorders [18].

In our study, the cutoff for neonatal and maternal complications was set at sAlb 3.2 mg/dL (Fig. S2). Since sAlb decreases gradually as pregnancy progresses (Table 2) [19], different cutoffs may apply to women in different settings. This study examined pregnancies among women on dialysis undergoing regular blood tests. However, for most women not receiving dialysis, obtaining biochemistry data in the first trimester may not be practical, as they are not necessarily recommended by guidelines to undergo such testing [20].

**Table 2:** Comparison of biochemistry, anthropometry findings and dialysis prescription in each trimester between preterm and full-term deliveries

	1st trimester			2nd trimester			3rd trimester		
	Preterm	Full-term	*P*-value	Preterm	Full-term	*P*-value	Preterm	Full-term	*P*-value
Number	9	5		9	5		5	5	
Age (years)	36.7 ± 3.5	33.6 ± 4.5	0.18						
Dialysis vintage (years)	10.5 ± 7.7	7.6 ± 4.9	0.47						
Biochemical parameters									
Timing of examination (weeks)^a^	13 ± 1	12 ± 3	0.49	21 ± 1	21 ± 1	0.35	33 ± 2	32 ± 2	0.70
Blood urea nitrogen (mg/dL)	45 ± 22	56 ± 11	0.29	38 ± 7	50 ± 14	0.053	34 ± 16	24 ± 10	0.26
Creatinine (mg/dL)	8.7 ± 2.5	9.7 ± 1.9	0.44	7.4 ± 1.0	9.0 ± 2.6	0.12	6.4 ± 2.7	5.8 ± 1.9	0.69
Albumin (g/dL)	3.3 ± 0.2	3.6 ± 0.3	0.09	3.1 ± 0.4	3.3 ± 0.2	0.18	2.7 ± 0.4	2.9 ± 0.1	0.37
Potassium (mEq/L)	4.3 ± 0.6	4.6 ± 0.9	0.50	4.1 ± 0.4	4.5 ± 0.6	0.18	3.9 ± 0.6	3.9 ± 0.7	0.88
Calcium (mg/dL)	8.8 ± 0.6	9.1 ± 0.6	0.41	9.1 ± 0.5	9.2 ± 0.2	0.54	8.7 ± 0.7	9.1 ± 0.6	0.29
Phosphate (mg/dL)	4.5 ± 1.8	5.2 ± 1.4	0.43	4.2 ± 1.0	3.9 ± 0.8	0.33	3.8 ± 1.5	3.6 ± 1.0	0.88
Hemoglobin (g/dL)	9.7 ± 0.9	10.0 ± 0.8	0.48	9.8 ± 1.7	10.0 ± 1.5	0.71	11.0 ± 1.5	9.8 ± 0.4	0.12
Blood pressure and dry weight									
Timing of examination (weeks)^a^	13 ± 1	12 ± 3	0.49	20 ± 3	21 ± 1	0.33	33 ± 2	32 ± 2	0.70
Systolic blood pressure (mmHg)	128 ± 32	122 ± 26	0.71	136 ± 29	117 ± 17	0.21	141 ± 21	115 ± 15	0.0496
Diastolic blood pressure (mmHg)	75 ± 16	72 ± 20	0.71	79 ± 21	69 ± 10	0.36	90 ± 12	68 ± 10	0.013
Dry weight (kg)	58.6 ± 10.8	60.6 ± 12.0	0.76	60.3 ± 11.4	62.0 ± 11.7	0.80	63.3 ± 12.8	65.6 ± 11.9	0.78
Dialysis prescription									
Dialysis time (hour/week)	19.8 ± 4.9	20.5 ± 9.9	0.86	22.5 ± 3.7	22.9 ± 8.7	0.91	24.8 ± 5.0	27.8 ± 5.2	0.38
Frequency (times/week)	4.2 ± 0.8	4.4 ± 1.3	0.76	5.1 ± 0.9	4.8 ± 1.3	0.67	5.4 ± 0.5	5.8 ± 0.4	0.24

^a^Gestational weeks

Neonatal complications occurred in 2 of 3 infants with extremely preterm delivery and in 1 of the 4 FGR infants. Despite having repeated HDP and FGR, Case 6 had second and third deliveries without neonatal complications at 37 and 36 weeks, respectively, after taking a low dose of aspirin (presumably following expert recommendations [21]) and increasing both the dialysis blood flow and the size of the dialysis membrane. Three pregnancies which occurred among women who began dialysis between the ages of 12 to 17 resulted in two extremely preterm deliveries and one delivery with placental abruption and severe fetal complications. These observations raise the possibility that kidney disease onset, immunosuppressive treatment and dialysis initiation during adolescence adversely affected the functional maturation of the uterus. Indeed, adolescent pregnancy in general is reported to be associated with maternal and neonatal complications [22].

The present study also provides some hints about the relationship between the cause of ESRD and pregnancy outcomes. A previous study indicated that ESRD due to glomerulonephritis was associated with increased live birth rates, while diabetes was associated with decreased live birth rates [23]. On the other hand, in a Canadian study by Hladunewich et al. among 17 women on dialysis, extremely preterm deliveries of 360 g and 980 g infants occurred in mothers who had ESRD due to IgA nephropathy [10]. Hoffman et al. reported that ESRD due to diabetes or lupus was associated with neonatal morbidity [24]. In our study, mean age at dialysis initiation among 4 mothers who had ESRD due to diabetic nephropathy (33.5 ± 3.9 years, range 29–38) was much older than that of the other 11 mothers (23.6 ± 7.4 years, range 12–39, *P* = 0.03). Our findings suggest that dialysis initiation at a relatively young age reduces the probability of developing ESRD due to diabetic nephropathy.

In our study, HDP (52.6%) and preterm delivery (64.3%) were frequent during pregnancy in women on dialysis (Table 1). In pregnancy in the general population, these factors are tightly linked, and are associated with increased risk for neonatal morbidity and mortality [18]. In our study, there were 3 women who did not have chronic hypertension at pregnancy start but developed HDP and preterm labor later (Table 1). Importantly, ideal BP target for hypertensive mothers is controversial [25, 26]. A recent study reported that initiating tight (versus less-tight) control of BP at < 24 weeks significantly reduced severe maternal hypertension and preterm birth (without a significant effect on maternal death). However, this effect was counterbalanced by an increased incidence of mildly small for gestational age, such that there was no overall effect on neonatal morbidity or death [27]. On the other hand, a randomized clinical trial in the general population revealed that aggressive treatment of mild to moderate hypertension in the third trimester reduced premature delivery and neonatal respiratory distress requiring intensive care compared to placebo treatment [28]. Taken together, the above findings suggest that the timing to potentiate antihypertensive treatment is an important consideration for pregnancy both in general and under conditions of dialysis.

It is well established that low BUN is associated with favorable maternal and neonatal outcomes in pregnancy with ESRD [9–11, 29]. However, BUN may not be a simple surrogate of uremic toxin accumulation [10]. Frequent, long-time dialysis should not only efficiently remove uremic toxins but also reduce the amount and speed of water removal during the dialysis session, which should be helpful for stabilizing maternal and fetal circulation. In that sense, BUN seems to be a reliable surrogate for dialysis performance. In a single-center, core facility report from Brazil [30], favorable fetal outcomes were obtained when midweek BUN was < 35 mg/dL. Our study consistently found that mean BUN at the beginning of the week in the third trimester was 34 mg/dL and 24 mg/dL (*P* = 0.26) in the preterm and full-term cases, respectively (Table 2). When women on dialysis become pregnant, it is recommended that they increase protein and total calorie intake from the second trimester to compensate for fetal growth and intensified dialysis-induced protein loss [31], but information about actual or recommended protein intake was not collected in our study. It is interesting to note that, in the second trimester, Case 6 showed phosphate levels of 5.2 ± 0.5 mg/dL in three pregnancies (6P1–6P3), which were higher than the overall mean level of 4.1 mg/dL among 14 pregnancies. This might have been caused by increased protein intake to maintain sAlb, by poor compliance in taking phosphate binders by the patient, or by the use of low efficiency dialysis to cope with dialysis difficulty, for instance, by intradialytic hypotension. Therefore, we have to point out there is the possibility that higher BUN level may not be a cause of poor pregnancy outcomes, but a result of difficult dialysis. This view is supported by a study by Luders et al. of pregnancy in women with ESRD [32]. In their study, the adverse fetal outcome group had higher BUN levels than the favorable fetal outcome group, but the dialysis doses were similar between the groups, indicating that low efficiency dialysis was not the cause of the poor outcomes.

In our survey, the live birth rate was 77.8%, and preterm delivery was predominant among these live births. However, there was no occurrence of maternal death or of congenital abnormalities. Importantly, although the live birth rate in women on dialysis is much lower than that in the general population (see below) [1, 29], a recent, large-scale, meta-analysis reported that ESRD patients have a maternal perinatal mortality rate of only 0.4% and the same congenital abnormality rate as the general population (2%) [11].

We further compared the results of the survey in 1996 [8] and this survey in 2017 (Table S3). The response rate in 1996 was 1.8-fold higher than that in 2017. It was difficult to compare the frequency of pregnancy (3.4% versus 0.25%/year), because the 1996 study did not specify the time period covered. Moreover, in the 2017 study, there were tendencies that mean gestational age was longer, the preterm delivery rate was lower, and the birth weight was higher, despite the fact that the mothers were older and the duration of dialysis prior to pregnancy was longer. The frequency of major neonatal complications in surviving infants, which were tightly associated with lower gestational age and birth weight (Fig. 1), decreased from 41.7 to 21.4% (*P* = 0.18). In addition, mean dialysis time before delivery increased from 22.0 to 24.9 h per week and mean dialysis frequency increased from 4.5 to 5.6 times per week (Table S3).

The tendency toward improved pregnancy outcomes over 21 years may have been caused by longer and more frequent dialysis [10, 11, 29], good anemia control (Table 2) [9], and technical progress in pregnancy and neonate care. Indeed, the rate of neonatal death in the Japanese general population improved from 2.0 per 1000 live born babies in 1996 [33] to 0.9 in 2016 [34].

We also compared the outcomes of pregnancy on dialysis among nationwide surveys in other countries published in 1998 or later (Table 3). The mean dialysis vintage of 8.4 years in our study was almost double that of the longest dialysis vintages previously reported for other countries [23, 35]. Our study found that the frequency of pregnancy was 2.5 per 1000 patient-years (PTPY) and the live birth rate was 78%. This fit within the ranges of 2.1–5.5 PTPY and 40–83% in studies from Belgium [36], the USA [10, 37], Australia [23], New Zealand [23] and Canada [10]. These findings are also consistent with a systematic review of 10 surveys published between 2000 and 2008 reporting that the mean live birth rate was 76% [38].

**Table 3:** Nationwide surveys concerning pregnancy on dialysis

References	First author	Country	Study period	Target population (cases)	Target age (years)	Mean age at pregnancy (years)	Mean dialysis vintage (years)	Pregnancy (times/patients)	Pregnancy rate (PTPY)	Surviving infant rate^a^ (%)
AJKD 1998 [37]	Okundaye	USA	1992–1995	6230 HD + PD	14–44	ND	1 to 5	135/ND	> 5.5	40
AJKD 1998 [36]	Bagon	Beigium	1975–1996	1472 HD + PD	18–44	28.0 ± 3.2	3.0	15/14	3.3	50
Nephrology 2013 [23]	Shahir	AU/NZ	1966–2008	7796 HD + PD	15–49	30.7	4.0	49/ND	2.1	79
JASN 2014 [10]	Hladunewich	USA	1990–2011	ND, HD	ND	27 ± 6	1.5	70/ND	ND	53
JASN 2014 [10]	Hladunewich	Canada	2000–2013	ND, HD	ND	34 ± 4	3.4	22/17	ND	83
JASN 2019 [39]	Shah	USA	2005–2013	47,555 HD + PD	15–44	29 ± 6	< 1	2352^b^/ND	17.8^c^	39
AJKD 2020 [35]	Oliverio	USA	2002–2015	12,382 HD	18–44	30.6	4.4	ND/664^b^	(2.1–3.6)^d^	ND
Tsubasa	Hirano	Japan	2012–2016	1992 HD	15–44	34.6 ± 5.7	8.4	25/20	2.5^e^	78

*PTPY* per thousand patient-years, *HD* hemodialysis, *PD* peritoneal dialysis, *AU/NZ* Australia and New Zealand, *ND* not described

^a^Among pregnant patients on dialysis, excluding unknown outcomes

^b^The study used administrative claim data, and may not be comparable to other survey-based cohort studies

^c^More than half of patients were on dialysis for less than a year at study entry, which should have affected pregnancy rate in the study

^d^Delivery rate (pregnancy rate was not described)

^e^Assuming that all patients received dialysis for 5 full years

Recently, a study in the USA using administrative claim data instead of survey data found that the delivery rate (but not pregnancy rate) in women undergoing hemodialysis increased from 2.1 to 3.6 PTPY, during the years 2002 to 2015 [35]. Another claim-based report in the USA covering 2005 to 2013 indicated that the pregnancy rate was 17.8 PTPY and the live birth rate was 27% (39% when unknown outcomes were excluded), but mean dialysis vintage was recorded as less than a year, suggesting that there was a reporting bias [39].

In our study, the incidence of live births was 1.4 PTPY among women on dialysis aged from 15 to 44 years (calculated as 14/1992/5), compared to 46.9 PTPY in the general Japanese population in 2016 (according to Vital Statistics collected by Ministry of Health, Labour and Welfare of Japan; 975,531 live births [40] among 20,814,958 women aged 15–44 [41]). A similar survey in Italy reported live birth rates of 0.7–1.1 PTPY for women on dialysis, and 72.5 PTPY for the general population aged 20–45 [1].

Our survey has several limitations. The study design is limited by the fact that a survey was used to determine pregnancy rates and complications, and by its low response rate, both of which may have led to underreporting. Also, this study is a retrospective case series and has a small number of events, so the statistical power of this study is restricted, and we cannot establish cause/effect relationships. However, a strength in this study is that all of the pregnant women belonged to independent units, except for one pair of women, reflecting a multi-center feature of our analysis.

## Conclusions

In conclusion, following up on a 1996 nationwide survey by Toma et al. [8], our 2017 study found a tendency toward improved pregnancy outcomes in Japanese women on hemodialysis. Surviving infants were successfully delivered even after 10 years of hemodialysis. The study suggests that ESRD caused by diabetic nephropathy and age at conception ≥ 38 years are potential risk factors for maternal complications. Compared to full-term cases, preterm cases showed a tendency toward lower birth weights, more frequent development of HDP, malnutrition, and older mothers with longer dialysis vintage. Furthermore, we observed that neonatal complications not only occurred as a result of extremely preterm delivery or FGR but also were predictively associated with malnutrition in the first trimester and with certain causes of ESRD in relatively young mothers. These findings may provide guidance to medical practitioners for treating both current ESRD patients and young patients who have not yet developed ESRD.

## Supplementary Information

Below is the link to the electronic supplementary material.

Supplementary file1 (DOCX 109 kb)

## Data Availability

The datasets used and/ or analyzed in the current study are available from the corresponding author upon reasonable request.

## References

[1] Piccoli GB, Cabiddu G, Daidone G, Guzzo G, Maxia S, Ciniglio I, Postorino V, Loi V, Ghiotto S, Nichelatti M, Attini R, Coscia A, Postorino M, Pani A, Italian Study Group on Kidney and Pregnancy (2014). The children of dialysis: live-born babies from on-dialysis mothers in Italy—an epidemiological perspective comparing dialysis, kidney transplantation and the overall population. Nephrol Dial Transplant.

[2] Network JOT (2017) The average waiting time for cadaveric renal transplantation (in Japanese). Organ Donation & Transplantation Data Book 2017:580. https://www.jotnw.or.jp/files/page/datas/databook/doc/15_kidneytx.pdf

[3] Transplantation TJSf (2017) The numbers of living and cadaveric renal transplantation (in Japanese). Fact book 2017 of Organ Transplantation in Japan:Table 2. http://www.asas.or.jp/jst/pdf/factbook/factbook2017.pdf

[4] Therapy TJSfD (2018) The numbers of patients who started hemodialysis or peritoneal dialysis (in Japanese). 2018 Annual Dialysis Data Report, JSDT Renal Data Registry:685. https://docs.jsdt.or.jp/overview/file/2018/pdf/01.pdf

[5] Goodkin DA, Bragg-Gresham JL, Koenig KG, Wolfe RA, Akiba T, Andreucci VE, Saito A, Rayner HC, Kurokawa K, Port FK, Held PJ, Young EW (2003). Association of comorbid conditions and mortality in hemodialysis patients in Europe, Japan, and the United States: the Dialysis Outcomes and Practice Patterns Study (DOPPS). J Am Soc Nephrol.

[6] Confortini P (1971) Full term successful pregnancy and successful delivery in a patient on chronic haemodialysis. In: Proc Eur Dial Transplant Assoc, pp 74–80

[7] Kobayashi H, Matsumoto Y, Otsubo O, Otsubo K, Naito T (1981). Successful pregnancy in a patient undergoing chronic hemodialysis. Obstet Gynecol.

[8] Toma H, Tanabe K, Tokumoto T, Kobayashi C, Yagisawa T (1999). Pregnancy in women receiving renal dialysis or transplantation in Japan: a nationwide survey. Nephrol Dial Transplant.

[9] Asamiya Y, Otsubo S, Matsuda Y, Kimata N, Kikuchi KAN, Miwa N, Uchida K, Mineshima M, Mitani M, Ohta H, Nitta K, Akiba T (2009). The importance of low blood urea nitrogen levels in pregnant patients undergoing hemodialysis to optimize birth weight and gestational age. Kidney Int.

[10] Hladunewich MA, Hou S, Odutayo A, Cornelis T, Pierratos A, Goldstein M, Tennankore K, Keunen J, Hui D, Chan CT (2014). Intensive hemodialysis associates with improved pregnancy outcomes: a Canadian and United States cohort comparison. J Am Soc Nephrol.

[11] Piccoli GB, Minelli F, Versino E, Cabiddu G, Attini R, Vigotti FN, Rolfo A, Giuffrida D, Colombi N, Pani A, Todros T (2016). Pregnancy in dialysis patients in the new millennium: a systematic review and meta-regression analysis correlating dialysis schedules and pregnancy outcomes. Nephrol Dial Transplant.

[12] Gynecology JSoOa, Gynecologists JAoOa (2020) CQ307-1: screening for fetal growth restriction (in Japanese). Obstetrics and gynecology practice guidelines: Obstetrics Edition 157–159. https://minds.jcqhc.or.jp/n/med/4/med0097/G0001189)

[13] Watanabe K, Matsubara K, Nakamoto O, Ushijima J, Ohkuchi A, Koide K, Makino S, Mimura K, Morikawa M, Naruse K, Tanaka K, Nohira T, Metoki H, Kawabata I, Takeda S, Seki H, Takagi K, Yamasaki M, Ichihara A, Kimura T, Saito S (2018). Outline of the new definition and classification of “Hypertensive Disorders of Pregnancy (HDP)”; a revised JSSHP statement of 2005. Hypertens Res Pregnancy.

[14] Engle WA, Tomashek KM, Wallman C, Committee on Fetus & Newborn AAoP (2007). "Late-preterm" infants: a population at risk. Pediatrics.

[15] Stoll BJ, Hansen NI, Bell EF, Shankaran S, Laptook AR, Walsh MC, Hale EC, Newman NS, Schibler K, Carlo WA, Kennedy KA, Poindexter BB, Finer NN, Ehrenkranz RA, Duara S, Sanchez PJ, O'Shea TM, Goldberg RN, Van Meurs KP, Faix RG, Phelps DL, Frantz ID, Watterberg KL, Saha S, Das A, Higgins RD, Kennedy Shriver National Institute of Child Health and Human Development Neonatal Research Network (2010). Neonatal outcomes of extremely preterm infants from the NICHD Neonatal Research Network. Pediatrics.

[16] Liu ZN, Cui Z, He YD, Zhang YM, Wang F, Wang X, Meng LQ, Cheng XY, Liu G, Zhao MH (2020). Membranous nephropathy in pregnancy. Am J Nephrol.

[17] Siligato R, Gembillo G, Cernaro V, Torre F, Salvo A, Granese R, Santoro D (2020). Maternal and fetal outcomes of pregnancy in nephrotic syndrome due to primary glomerulonephritis. Front Med (Lausanne).

[18] Villar J, Carroli G, Wojdyla D, Abalos E, Giordano D, Ba'aqeel H, Farnot U, Bergsjo P, Bakketeig L, Lumbiganon P, Campodonico L, Al-Mazrou Y, Lindheimer M, Kramer M, World Health Organization Antenatal Care Trial Research Group (2006). Preeclampsia, gestational hypertension and intrauterine growth restriction, related or independent conditions?. Am J Obstet Gynecol.

[19] Abduljalil K, Furness P, Johnson TN, Rostami-Hodjegan A, Soltani H (2012). Anatomical, physiological and metabolic changes with gestational age during normal pregnancy: a database for parameters required in physiologically based pharmacokinetic modelling. Clin Pharmacokinet.

[20] Gynecology JSoOa, Gynecologists JAoOa (2020) CQ003: Blood test items recommended in early pregnancy (in Japanese). Obstetrics and gynecology practice guidelines: Obstetrics Edition: 6–7. https://minds.jcqhc.or.jp/n/med/4/med0097/G0001189)

[21] LeFevre ML, Force USPST (2014). Low-dose aspirin use for the prevention of morbidity and mortality from preeclampsia: U.S. Preventive Services Task Force recommendation statement. Ann Intern Med.

[22] Conde-Agudelo A, Belizan JM, Lammers C (2005). Maternal-perinatal morbidity and mortality associated with adolescent pregnancy in Latin America: Cross-sectional study. Am J Obstet Gynecol.

[23] Shahir AK, Briggs N, Katsoulis J, Levidiotis V (2013). An observational outcomes study from 1966–2008, examining pregnancy and neonatal outcomes from dialysed women using data from the ANZDATA Registry. Nephrology (Carlton).

[24] Hoffman M, Sibai B (2020). Dialysis in pregnancy: role of the underlying cause of renal failure on peripartum outcomes. Am J Perinatol.

[25] Magee LA, von Dadelszen P, Rey E, Ross S, Asztalos E, Murphy KE, Menzies J, Sanchez J, Singer J, Gafni A, Gruslin A, Helewa M, Hutton E, Lee SK, Lee T, Logan AG, Ganzevoort W, Welch R, Thornton JG, Moutquin JM (2015). Less-tight versus tight control of hypertension in pregnancy. N Engl J Med.

[26] Abalos E, Duley L, Steyn DW, Gialdini C (2018). Antihypertensive drug therapy for mild to moderate hypertension during pregnancy. Cochrane Database Syst Rev.

[27] Pels A, Mol BWJ, Singer J, Lee T, von Dadelszen P, Ganzevoort W, Asztalos E, Magee LA, Group CS (2018). Influence of gestational age at initiation of antihypertensive therapy: secondary analysis of CHIPS trial data (control of hypertension in pregnancy study). Hypertension.

[28] Phippard AF, Fischer WE, Horvath JS, Child AG, Korda AR, Henderson-Smart D, Duggin GG, Tiller DJ (1991). Early blood pressure control improves pregnancy outcome in primigravid women with mild hypertension. Med J Aust.

[29] Cabiddu G, Castellino S, Gernone G, Santoro D, Giacchino F, Credendino O, Daidone G, Gregorini G, Moroni G, Attini R, Minelli F, Manisco G, Todros T, Piccoli GB, Italian Study Group on Kidney and Pregnancy (2015). Best practices on pregnancy on dialysis: the Italian Study Group on Kidney and Pregnancy. J Nephrol.

[30] Luders C, Titan SM, Kahhale S, Francisco RP, Zugaib M (2018). Risk factors for adverse fetal outcome in hemodialysis pregnant women. Kidney Int Rep.

[31] Esposito P, Garibotto G, Picciotto D, Costigliolo F, Viazzi F, Conti NE (2020). Nutritional challenges in pregnant women with renal diseases: relevance to fetal outcomes. Nutrients.

[32] Luders C, Castro MC, Titan SM, De Castro I, Elias RM, Abensur H, Romao JE (2010). Obstetric outcome in pregnant women on long-term dialysis: a case series. Am J Kidney Dis.

[33] Ministry of Health LaWoJ (2011) The rate of neonatal death in Japanese general population in 1996 (in Japanese). Report of vital statistics: Table 2–2. https://www.mhlw.go.jp/toukei/saikin/hw/jinkou/geppo/nengai11/toukei02.html

[34] Ministry of Health LaWoJ (2016) The rate of neonatal death in Japanese general population in 2016 (in Japanese). Report of vital statistics. https://www.mhlw.go.jp/toukei/saikin/hw/jinkou/kakutei16/dl/03_h1.pdf

[35] Oliverio AL, Bragg-Gresham JL, Admon LK, Wright Nunes JA, Saran R, Heung M (2020). Obstetric deliveries in US women with ESKD: 2002–2015. Am J Kidney Dis.

[36] Bagon JA, Vernaeve H, De Muylder X, Lafontaine JJ, Martens J, Van Roost G (1998). Pregnancy and dialysis. Am J Kidney Dis.

[37] Okundaye I, Abrinko P, Hou S (1998). Registry of pregnancy in dialysis patients. Am J Kidney Dis.

[38] Piccoli GB, Conijn A, Consiglio V, Vasario E, Attini R, Deagostini MC, Bontempo S, Todros T (2010). Pregnancy in dialysis patients: is the evidence strong enough to lead us to change our counseling policy?. Clin J Am Soc Nephrol.

[39] Shah S, Christianson AL, Meganathan K, Leonard AC, Schauer DP, Thakar CV (2019). Racial differences and factors associated with pregnancy in ESKD patients on dialysis in the United States. J Am Soc Nephrol.

[40] Ministry of Health LaWoJ (2016) The number of live-born babies in Japan (in Japanese). Report of vital statistics. https://www.mhlw.go.jp/toukei/saikin/hw/jinkou/kakutei16/dl/08_h4.pdf

[41] Ministry of Health LaWoJ (2016) The number of women aged 15–44 (in Japanese) Report of vital statistics. https://www.mhlw.go.jp/toukei/saikin/hw/jinkou/kakutei16/dl/13_fu.pdf

