# Supplementary material for: Pregnancy and delivery in women receiving maintenance hemodialysis in Japan: analysis of potential risk factors for neonatal and maternal complications
Source: J Nephrol. 2021 Sep 30;34(5):1599–609. doi: 10.1007/s40620-021-01146-3 (PMC8494660; doi:10.1007/s40620-021-01146-3)
Supplement: Supplementary file 1 — Supplementary file1 (DOCX 109 kb) [file 40620_2021_1146_MOESM1_ESM.docx]

**Electronic supplementary material**

**Pregnancy and delivery in women receiving maintenance hemodialysis in Japan: analysis of potential risk factors for neonatal and maternal complications**

Hiroko Hirano, Tomomi Ueda, Hirohiko Tani, Kenzo Kosaka, Eiji Nakatani,

Philip Hawke, Kiyoshi Mori, Noriko Mori


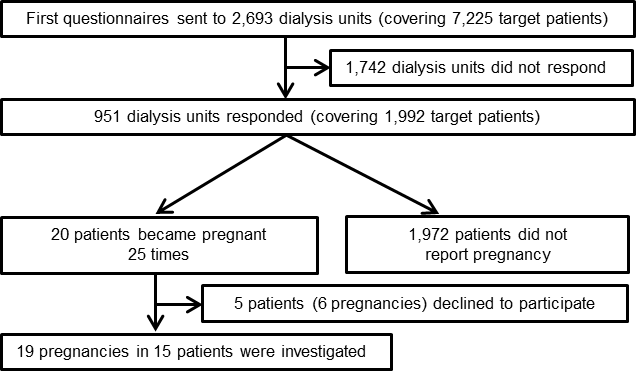


**Fig. S1. Survey flow chart**

**
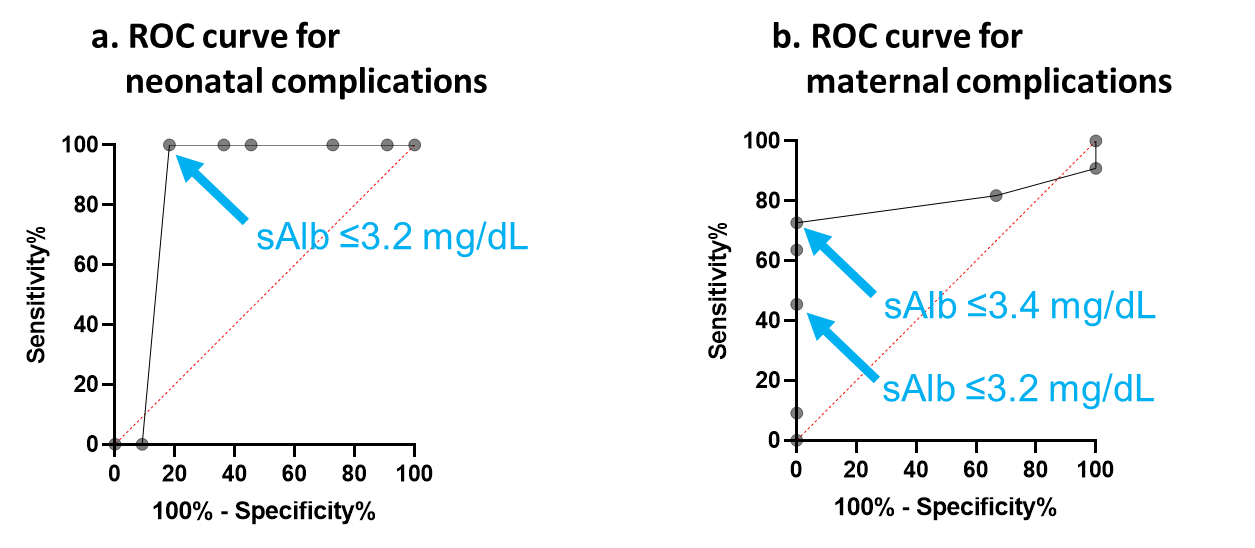
**

**Fig. S2. ROC curve analysis for (a) neonatal and (b) maternal complications in 14 surviving infants**

Area under the curve was (a) 0.864 (P = 0.062) and (b) 0.803 (P = 0.120), respectively. With a cutoff level of serum albumin (sAlb) ≤3.2 mg/dL, sensitivity and specificity for neonatal complications were 100% and 82%, and those for maternal complications were 45% and 100%, respectively.

**Table S1. Maternal and neonatal complications in pregnant women on dialysis and potential related risk factors**

*sAlb* serum albumin at the first trimester, dialysis vintage (blue, ≤1; yellow, 10-20; red, >20 years), *CS* caesarean section, *GA* gestational age shown as weeks+days (green, ≥37; red, <28 weeks), *BW* birth weight (red, <-1.5SD), *P1-P3* first, second, and third pregnancy, *V* vaginal delivery, *cHT* chronic hypertension at pregnancy start, *HDP* hypertensive disorders of pregnancy (orange), *sHT* severe hypertension during pregnancy (red), maternal complications not including HDP (yellow), *NRFS* non-reassuring fetal status, *FGR* fetal growth restriction, *PROM* pre-mature rupture of membranes, *Surviving* surviving infant, *S. abortion* spontaneous abortion (yellow), *E. abortion* elective abortion (blue), *RDS* respiratory distress syndrome, *ASD* atrial septal defect, *VSD* ventricular septal defect.

**Table S2. Biochemical parameters, blood pressure, dry weight, and dialysis prescription in each trimester**

^a^Gestational weeks

**Table S3. Comparison of Japanese nationwide surveys in 1996 and this survey in 2017 concerning pregnancy on dialysis**

(Legend for Table S3)

^a^Target patients were women aged 15-44 years with ESRD undergoing dialysis.

^b^Live birth rate was calculated, except for cases with elective abortion or unknown outcome.

^c^Maximal dialysis prescription before delivery.

^d^Gestational age and birth weight were evaluated, except for cases with spontaneous abortion, elective abortion, or unknown outcome.

*SD* standard deviation, *S* surviving infant, *N* neonatal death, *C* major fetal complication in surviving infant.
